# Influence of Tobacco upon the Circulatory System

**Published:** 1869-08

**Authors:** 


Influence of Tobacco upon the Circulatory System. Dr. Decaisne, in the course of investigation on the influence of tobacco on the circulation, has been struck with the large number of boys, aged from nine to fifteen years, who smoke, and has been led to inquire into the connection of this habit with impairment of the general health. He has observed 33 boys, aged from nine to fifteen, who smoke more or less. Of these, distinct symptoms were present in 27. In 22 there were various disorders of the circulation—*bruit de souffle* in the neck, palpitation, disorders of digestion, slowness of intellect, and a more or less marked taste for strong drinks. In three the pulse was intermittent. In eight there was found on examination more or less diminution of the red corpuscles; in twelve there was rather frequent epistaxis; ten had disturbed sleep, and four had slight ulcerations of the mucous membrane of the mouth, which disappeared on ceasing the use of tobacco for some days. In children who were well nourished the disorder was, in general, less marked. As to the ages, eight of the boys were from 9 to 12 years old; 19, 12 to 15. The duration of the habit of smoking was, in 11, from six months to a year, and in 15 more than two years. Ordinary treatment of anæmia in general produced no effect as long as the smoking was continued; but when this was desisted from health was soon perfectly restored, if there was no organic disease.

